# A spatiotemporal database on the energy, macro and micro-nutrients from the Brazilian agricultural production

**DOI:** 10.1016/j.dib.2020.105602

**Published:** 2020-04-22

**Authors:** João Pompeu, Jacqueline Gerage, Jean Ometto

**Affiliations:** Earth System Science Centre/National Institute for Space Research (CCST/INPE), São José dos Campos, Brazil

**Keywords:** Food and nutrition security, Land use, Nutritional yield, Geographic analysis, Agricultural political economy, Nutrition sensitive

## Abstract

To construct this database, we integrate the nutritional content of 62 crops and 5 livestock categories to estimate the amount of 21 macro and micro-nutrients (including energy) that were produced from agriculture in each Brazilian municipality during the last three decades. Additionally, we allocate these nutrients according to their share in the food system (for example, human food, animal feed, export etc.). It is a unique data source on macro and micro-nutrients availability for human consumption and animal feed, but also regarding another aspects of the food system, such as international agricultural trade, energy production (for example, in the form of ethanol) or post-harvest and post-processing losses, from local to national levels, in a wide time frame of 30 years. This database can be used in scientific research regarding food and nutrition security and in the construction of indicators for monitoring food and agricultural programs and policies that aim at the promotion of food and nutritional security. Also, it has the potential to enable broader analysis of the food system as whole in terms of food stability and resilience.

Specifications table

**Table:** 

Subject	Agricultural and Biological Sciences (General),
Specific subject area	Land use and food security
Type of data	Table
How data were acquired	We acquired subnational crop and livestock production from the National Institute for Geography and Statistics (IBGE); nutritional values of the agricultural commodities were taken from the Brazilian Table of Food Composition (TACO) whenever they were available, otherwise we used The United States Department of Agriculture (USDA)'s National Food and Nutrient Analysis Program (NFNAP) values; and agricultural products use in the food system from the Food and Agriculture Organization's (FAO) Food Balance Sheets (FBS).
Data format	Raw
Parameters for data collection	We collected the production of 62 crops and 5 livestock categories that were available for all of the 5563 Brazilian municipalities (the lowest administrative level) between 1988 and 2017.
Description of data collection	Crop and livestock production was collected from the National Institute for Geography and Statistics (IBGE) and transformed to nutrient equivalents based on the nutritional values of each agricultural commodity. Then, we calculated the proportion of each nutrient that is either delivered to the food system or lost based on the FAO's Food balance Sheets.
Data source location	All the 5563 Brazilian municipalities (specified with a unique geocode in the tables)
Data accessibility	Repository name: Pangaea Data identification number: 22771 Direct URL to data: https://doi.pangaea.de/10.1594/PANGAEA.911574

## Value of the data

• This dataset has the potential to be used in scientific research about spatio-temporal dynamics of agricultural production and for monitoring the broader aspects of food systems, food and nutrition security policies and governance from local to national levels in a considerable time frame.
• The scientific community that is interested in food system dynamics and food security may be directly benefited; local, regional and national stakeholders in the field of agricultural production, food governance and nutrition are also potential beneficiaries of this dataset.
• It can be used to build food security and nutrition indicators, to map regions with possible prevalence of food insecurity and to aid the analysis of spatio-temporal aspects of the Brazilian food system (e.g. stability and resilience studies).
• This data reports for the first time how much macro and micro-nutrients were produced from agriculture in all the Brazilian municipalities along the last tree decades. More considerably is the allocation of the nutrients human food, animal feed, commodity exports, losses etc.
• This dataset is useful for mapping spatial patterns of food production and their changes through time, indicating the most important regions for food production in the country.

## 1 Data Description

We provide a yearly dataset of 20 macro and micro-nutrients, plus energy (in kcal), derived from agricultural production (listed in the columns of Table 1), as well as their allocation in the Brazilian food system, to all of the 5563 Brazilian municipalities in the period of 1988 to 2017. Agricultural production was collected from the National Institute for Geography and Statistics (IBGE), the official source of statistics for Brazil. Five livestock and 62 crop products were available covering both the entire time series of 30 years and the whole country, so the complete agricultural information from IBGE was used in this database.

**Table 1:** IBGE names of the crops/livestock products, FAO Code as reported by the IBGE's PRODLIST, names of agricultural products in the FAO's FBS and values of all the 21 energy, macro and micro-nutrients used as reference for this database (in grams (g), milligrams (mg) or micrograms (ug) per 100 grams of product).

Crop/Livestock (IBGE)	FAO Equivalent Codes (PRODLIST)	FBS Equivalent names	Energy (kcal)	
Abacate	572	Fruits, other	96	
Abacaxi	574	Pineapples and products	48	
AlgdArb	328	Cottonseed	0	
AlgdHrb	328	Cottonseed	0	
Alho	406	Vegetables, Other	113	
Amendoim	242	Groundnuts	544	
Arroz	27	Rice	358	
Aveia	75	Oats	394	
Azeitona	260	Olive Olive Oil	137	
Banana	486	Bananas	92	
Batata doce	122	Sweet potatoes	118	
Batata inglesa	116	Potatoes and products	64	
Borracha	0837/0836		0	
Cacau	661	Cocoa Beans and products	74	
Café	656	Coffee and products	9	
Cana	156	Sugar cane	65	
Caqui	587	Fruits, other	71	
Castanha de caju	217	Nuts and products	553	
Cebola	402	Onions	39	
Centeio	71	Rye and products	338	
Cevada	44	Barley and products	354	
Chá	667	Tea (including mate)	2	
Coco da bahia	249	Coconuts - Incl Copra	406	
Dende	254	Coconut oil	884	
Erva Mate	671	Tea (including mate)	0	
Ervilha	187	Peas	88	
Fava	181	Pulses and other products	341	
Feijão	176	Beans	329	
Figo	569	Fruits, other	41	
Fumo	826		0	
Goiaba	603	Fruits, other	54	
Guaraná	603	Fruits, other	0	
Juta	780	Fiber	0	
Laranja	490	Oranges, mandarines	37	
Limão	497	Lemons, limes and products	32	
Linho	771	Fiber	0	
Maçã	515	Apples and products	56	
Malva	782	Fiber	0	
Mamão	600	Fruits, other	45	
Mamona	265	Oilcrops, other	0	
Mandioca	125	Cassava and products	151	
Manga	571	Fruits, other	64	
Maracujá	603	Fruits, other	68	
Marmelo	523		57	
Melancia	567	Fruits, other	33	
Melão	568	Fruits, other	29	
Milho	56	Maize and products	138	
Noz	234	Nuts and products	620	
Palmito	460		29	
Pera	521	Fruits, other	53	
Pessego	534	Fruits, other	36	
Pimenta do Reino	687	Pepper	251	
Rami	821	Fiber	0	
Sisal	789	Fiber	0	
Soja	236	Soybeans	446	
Sorgo	83	Sorghum and products	0	
Tangerina	495	Oranges, mandarines	38	
Tomate	388	Tomatoes and products	15	
Trigo	15	Wheat and products	198	
Tungue	275	Nuts and products	0	
Urucum	723	Spices, other	0	
Uva	560	Grapes and products (excl wine)	53	
Ovos	1067	Eggs	143	
Bovino	866	Bovine Meat	194.867	
Suínos	1034	Pigmeat	267.5	
Galináceos	1057	Poultry Meat	170.444	
Leite	882	Milk - Excluding Butter	67	
Óleo		Soybeans Oil	884	
Açúcar		Sugar	387	
Crop/Livestock (IBGE)	Carbohydrates (CHO) (g)	Fibre (NSP) (g)	Protein (g)	

Abacate	6	6.3	1.2	
Abacaxi	12.3	1	0.9	
AlgdArb	0	0	0	
AlgdHrb	0	0	0	
Alho	23.9	4.3	7	
Amendoim	20.3	8	27.2	
Arroz	78.8	1.6	7.2	
Aveia	66.6	9.1	13.9	
Azeitona	4.1	3.8	0.9	
Banana	23.8	1.9	1.4	
Batata doce	28.2	2.6	1.3	
Batata inglesa	14.7	1.2	1.8	
Borracha	0	0	0	
Cacau	19.4	2.2	1	
Café	1.5	0	0.7	
Cana	18.2	0.1	0	
Caqui	19.3	6.5	0.4	
Castanha de caju	30.19	3.3	18.22	
Cebola	1.7	2.2	1.7	
Centeio	75.86	15.1	10.34	
Cevada	73.48	17.3	12.48	
Chá	0.6	0	0	
Coco da bahia	10.4	5.4	3.7	
Dende	0	0	0	
Erva Mate	0	0	0	
Ervilha	14.2	9.7	7.5	
Fava	58.29	25	26.12	
Feijão	61.2	18.4	20	
Figo	10.2	1.8	1	
Fumo	0	0	0	
Goiaba	13	6.2	1.1	
Guaraná	0	0	0	
Juta	0	0	0	
Laranja	8.9	0.8	1	
Limão	11.1	1.2	0.9	
Linho	0	0	0	
Maçã	15.2	1.3	0.3	
Malva	0	0	0	
Mamão	11.6	1.8	0.8	
Mamona	0	0	0	
Mandioca	36.2	1.9	1.1	
Manga	16.7	2.1	0.4	
Maracujá	12.3	1.1	2	
Marmelo	15.3	1.9	0.4	
Melancia	8.1	0.1	0.9	
Melão	7.5	0.3	0.7	
Milho	28.6	3.9	6.6	
Noz	18.4	7.2	14	
Palmito	5.5	2.6	2.5	
Pera	14	3	0.6	
Pessego	9.3	1.4	0.8	
Pimenta do Reino	63.95	25.3	10.39	
Rami	0	0	0	
Sisal	0	0	0	
Soja	30.16	9.3	36.49	
Sorgo	0	0	0	
Tangerina	9.6	0.9	0.8	
Tomate	3.1	1.2	1.1	
Trigo	42.53	2.35	7.49	
Tungue	0	0	0	
Urucum	0	0	0	
Uva	13.6	0.9	0.7	
Ovos	1.6	0	13	
Bovino	1.007	0.04	19.707	
Suínos	0	0	18.162	
Galináceos	0	0	18.378	
Leite	4.58	0	3.33	
Óleo	0	0	0	
Açúcar	100	0	0.3	
Crop/Livestock (IBGE)	Potassium (K) (mg)	Calcium (Ca) (mg)	Magnesium (Mg) (mg)	Phosphorus (P) (mg)

Abacate	206	8	15	22
Abacaxi	131	22	18	13
AlgdArb	0	0	0	0
AlgdHrb	0	0	0	0
Alho	535	14	21	149
Amendoim	580	0	171	407
Arroz	62	4	30	104
Aveia	336	48	119	153
Azeitona	20	46	4	5
Banana	376	3	28	27
Batata doce	340	21	17	36
Batata inglesa	302	4	15	39
Borracha	0	0	0	0
Cacau	72	12	25	9
Café	156	3	10	9
Cana	18	9	12	5
Caqui	164	18	9	18
Castanha de caju	660	37	292	593
Cebola	176	14	12	38
Centeio	510	34	110	332
Cevada	452	33	133	264
Chá	13	2	1	2
Coco da bahia	354	6	51	118
Dende	0	0	0	0
Erva Mate	0	0	0	0
Ervilha	311	24	42	152
Fava	1062	103	192	421
Feijão	1352	123	210	385
Figo	174	27	11	15
Fumo	0	0	0	0
Goiaba	198	4	7	15
Guaraná	0	0	0	0
Juta	0	0	0	0
Laranja	163	22	9	23
Limão	128	51	10	24
Linho	0	0	0	0
Maçã	75	2	2	9
Malva	0	0	0	0
Mamão	222	25	17	11
Mamona	0	0	0	0
Mandioca	208	15	44	29
Manga	148	12	8	9
Maracujá	338	5	28	51
Marmelo	197	11	8	17
Melancia	104	8	10	12
Melão	216	3	6	10
Milho	185	2	33	113
Noz	533	105	153	396
Palmito	206	32	25	55
Pera	116	8	6	12
Pessego	124	3	4	15
Pimenta do Reino	1329	443	171	158
Rami	0	0	0	0
Sisal	0	0	0	0
Soja	1797	277	280	704
Sorgo	0	0	0	0
Tangerina	131	13	8	12
Tomate	222	7	11	20
Trigo	169	28	82	200
Tungue	0	0	0	0
Urucum	0	0	0	0
Uva	162	8	5	12
Ovos	150	42	13	164
Bovino	276.2	13.46	17.333	155.133
Suínos	8.667	18	151	0.725
Galináceos	242.444	11.25	26	186.222
Leite	133	134	10	82
Óleo	0	0	0	0
Açúcar	6	4	1	0
Crop/Livestock (IBGE)	Iron (Fe) (mg)	Copper (Cu) (mg)	Zinc (Z) (mg)	Manganese (mn) (mg)

Abacate	0.2	0.15	0.2	0.17
Abacaxi	0.3	0.11	0.1	1.62
AlgdArb	0	0	0	0
AlgdHrb	0	0	0	0
Alho	0.8	0.15	0.8	0.24
Amendoim	2.5	0.78	3.2	1.96
Arroz	0.7	0.11	1.2	1.03
Aveia	4.4	0.44	2.6	1.89
Azeitona	0.2	0.14	0.1	0.03
Banana	0.3	0.1	0.2	0.14
Batata doce	0.4	0.11	0.2	0.18
Batata inglesa	0.4	0.09	0.2	0.1
Borracha	0	0	0	0
Cacau	0.3	0.15	0.6	0.04
Café	0	0.01	0	0.04
Cana	0.8	0.01	0.1	0.21
Caqui	0.1	0	0.2	0.39
Castanha de caju	6.68	0	5.78	292
Cebola	0.2	0.05	0.2	0.13
Centeio	2.63	0	2.65	0
Cevada	3.6	0	2.77	0
Chá	0	0	0	0.09
Coco da bahia	1.8	0.45	0.9	1
Dende	0.01	0	0	0
Erva Mate	0	0	0	0
Ervilha	1.4	0.2	1.2	0.4
Fava	6.7	0	3.14	0
Feijão	8	0.79	2.9	1.02
Figo	0.2	0.13	0.1	0.06
Fumo	0	0	0	0
Goiaba	0.2	0.04	0.1	0.09
Guaraná	0	0	0	0
Juta	0	0	0	0
Laranja	0.1	0.03	0.1	0.05
Limão	0.2	0.06	0.2	0.07
Linho	0	0	0	0
Maçã	0.1	0.06	0	0.03
Malva	0	0	0	0
Mamão	0.2	1.36	0.1	0.04
Mamona	0	0	0	0
Mandioca	0.3	0.07	0.2	0.05
Manga	0.1	0.1	0.1	0.17
Maracujá	0.6	0.19	0.4	0.12
Marmelo	0.7	0	0.04	0
Melancia	0.2	0.04	0.1	0.14
Melão	0.2	0.04	0.1	0.05
Milho	0.4	0.05	0.5	0.12
Noz	2	0.75	2.1	4.05
Palmito	0.2	0.08	0.4	0.14
Pera	0.1	0.07	0.1	0.04
Pessego	0.2	0.02	0.1	0.05
Pimenta do Reino	9.71	0	1.19	0
Rami	0	0	0	0
Sisal	0	0	0	0
Soja	15.7	0	4.89	0
Sorgo	0	0	0	0
Tangerina	0.1	0.03	0	0.04
Tomate	0.2	0.04	0.1	0.07
Trigo	2.14	0	1.65	0
Tungue	0	0	0	0
Urucum	0	0	0	0
Uva	0.1	0.11	0	0.13
Ovos	1.6	0.06	1.1	0
Bovino	1.54	0.046	3.5	0.005
Suínos	0.08	1.495	0	0
Galináceos	0.622	0.038	1.333	0.013
Leite	0	0.02	0.4	0
Óleo	0	0	0	0
Açúcar	0.1	0	0	0
Crop/Livestock (IBGE)	Vit A (RAE) (ug)	Thiamin (mg)	Riboflavin (mg)	Niacin (mg)

Abacate	7	0	0.04	0
Abacaxi	3	0.17	0.02	0
AlgdArb	0	0	0	0
AlgdHrb	0	0	0	0
Alho	0	0.18	0	0
Amendoim	0	0.1	0.03	10.18
Arroz	0	0.16	0	1.12
Aveia	0	0.55	0.03	4.47
Azeitona	20	0	0	0
Banana	7	0	0.02	0
Batata doce	709	0.06	0	0
Batata inglesa	0	0.1	0	0
Borracha	0	0	0	0
Cacau	0	0.25	0	0
Café	0	0	0	0
Cana	0	0	0	0
Caqui	81	0	0	0
Castanha de caju	0	423	58	1062
Cebola	0	0.04	0	0
Centeio	1	316	251	4.27
Cevada	1	646	285	4604
Chá	0	3.11	0.48	0
Coco da bahia	0	0	0	0
Dende	0	0	0	0
Erva Mate	0	0	0	0
Ervilha	38	0.27	0.07	1.16
Fava	3	555	333	2832
Feijão	0	0.17	0	4.02
Figo	7	0.05	0	0
Fumo	0	0	0	0
Goiaba	31	0	0	0
Guaraná	0	0	0	0
Juta	0	0	0	0
Laranja	0	0.07	0.02	0
Limão	1	0.3	0.04	0
Linho	0	0	0	0
Maçã	2	0	0	0
Malva	0	0	0	0
Mamão	74	0.03	0.03	0
Mamona	0	0	0	0
Mandioca	1	0	0	0
Manga	54	0.02	0.06	0
Maracujá	64	0	0.05	0
Marmelo	2	0.02	0.03	0.2
Melancia	28	0	0	0
Melão	3	0	0	0
Milho	16	0.3	0	0
Noz	1	0.38	0	1.08
Palmito	0	0.03	0	0
Pera	1	0	0	0
Pessego	16	0.05	0	0
Pimenta do Reino	27	108	0.18	1143
Rami	0	0	0	0
Sisal	0	0	0	0
Soja	1	874	0.87	1623
Sorgo	0	0	0	0
Tangerina	34	0.06	0.02	0
Tomate	42	0.12	0	0
Trigo	0	225	155	3087
Tungue	0	0	0	0
Urucum	0	0	0	0
Uva	3	0	0	0
Ovos	160	0.07	0.58	0.75
Bovino	3.58	0.141	0.105	2.639
Suínos	7.5	0.046	6.438	0.033
Galináceos	34.25	0.116	0.034	3.401
Leite	125	0.4	0.24	1.52
Óleo	0	0	0	0
Açúcar	0	0	0	0
Crop/Livestock (IBGE)	B6 (mg)	VitaminC (mg)	VitB12 (ug)	Folato (ug)

Abacate	0	8.7	0	81
Abacaxi	0	34.6	0	18
AlgdArb	0	0	0	0
AlgdHrb	0	0	0	0
Alho	0.44	0	0	3
Amendoim	0.76	0	0	240
Arroz	0.07	0	0	8
Aveia	0	1.4	3	327
Azeitona	0	0	0	0
Banana	0.14	5.9	0	20
Batata doce	0.1	16.5	0	11
Batata inglesa	0.15	31.1	0	17
Borracha	0	0	0	0
Cacau	0.04	13.6	0	32
Café	0	0	0	0
Cana	0.03	2.8	0	0
Caqui	0.03	29.6	0	8
Castanha de caju	417	0.5	0	25
Cebola	0.14	4.7	0	19
Centeio	294	0	0	38
Cevada	318	0	0	19
Chá	0	0	0	0
Coco da bahia	0.03	2.5	0	26
Dende	0	0	0	0
Erva Mate	0	0	0	0
Ervilha	0.06	12.4	0	65
Fava	366	1.4	0	423
Feijão	0.65	0	0	525
Figo	0	0.8	0	6
Fumo	0	0	0	0
Goiaba	0.03	80.6	0	49
Guaraná	0	0	0	0
Juta	0	0	0	0
Laranja	0.02	53.7	0	0
Limão	0	38.2	0	11
Linho	0	0	0	0
Maçã	0.03	2.4	0	0
Malva	0	0	0	0
Mamão	0	78.5	0	0
Mamona	0	0	0	0
Mandioca	0.04	16.5	0	27
Manga	0.05	17.4	0	0
Maracujá	0.05	19.8	0	0
Marmelo	0.04	15	0	3
Melancia	0	6.1	0	3
Melão	0.02	8.7	0	8
Milho	0.04	0	0	42
Noz	0.13	0	0	98
Palmito	0	8.7	0	39
Pera	0	2.8	0	0
Pessego	0	3.3	0	4
Pimenta do Reino	291	0	0	17
Rami	0	0	0	0
Sisal	0	0	0	0
Soja	377	6	0	375
Sorgo	0	0	0	0
Tangerina	0.02	48.8	0	0
Tomate	0.02	21.2	0	15
Trigo	265	2.6	0	38
Tungue	0	0	0	0
Urucum	0	0	0	0
Uva	0.03	3.3	0	0
Ovos	0	0	0.89	47
Bovino	0.031	0	2.31	4
Suínos	0	2.5	17.25	0.385
Galináceos	0	0	0.47	4.375
Leite	0	0	0.45	0
Óleo	0	0	0	0
Açúcar	0	0	0	0

The dataset consists in 20 tables, each containing the values for a specific nutrient produced every year (columns) in the municipalities (rows). The tables are labelled with the name of the nutrient (e.g.: dataset_Energy.tab, dataset_Mg.tab...). Each municipality contains 8 rows corresponding to the allocation of the nutrient in the Brazilian food system: 1) the total amount of that nutrient produced in a specific year, based on nutritional information of 62 crops and 5 livestock categories; 2) the amount of a nutrient that was derived from crop processing to oil; 3) the quantity of nutrient delivered to the food system directly as food for human consumption; 4) the quantity of nutrient that was exported, thus, not available to the national food system; 5) the quantity of nutrient delivered to the food system as animal feed; 6) the quantity of nutrient that returned to the food system in the form of seeds; 7) other uses (e.g. ethanol production) and; 8) the amount of nutrients lost in post-harvesting and post-processing.

The names and the content of the columns are the following:

- geocod: the unique geocode of each Brazilian municipality, as defined by the National Institute of Geography and Statistics (IBGE). It can be used to transform the sheet to a shapefile.
- Municipality: the name of the municipality (State abbreviation).
- Type: which of the 20 nutrients listed in the Table 1 is reported in the sheet (with unit).
- FBS: total production of the nutrient in the municipality and the fraction that is delivered to the food system, from the FAO's Food Balance Sheet. Total means all of the produced nutrients; Processed means the fraction that is processed, mainly into oil; Food is the total amount of nutrient from food, after accounting for commodity processing; Export is the amount of exported nutrients after accounting for commodity processing; Feed is the amount of nutrients used for animal feed, after accounting for commodity processing; Seed is the amount of nutrients that return to the food system in the form of seeds; Other use is the amount of nutrients that are not delivered to the Brazilian food system, but domestically used, mainly in the form of Ethanol; Losses is the amount of nutrients that are lost from the food system after cropping and after processing.
- XYYYY: The years from 1988 to 2017 (e.g. X1988, X1989, X1990… X2017).

## 2 Experimental Design, Materials, and Methods

We generated the database on nutrients production and allocation in the Brazilian food system by converting the subnational agricultural production to nutrients equivalent, based on the nutrient composition of each agricultural commodity, following previous studies with the same methods [1,2].

### 2.1 Nutritional composition of the agricultural commodities

First, we collected 62 crop and 5 livestock categories production available for all of the 5563 Brazilian municipalities in the period of 1988 to 2017. This data is distributed by the IBGE and available in: https://sidra.ibge.gov.br/pesquisa/ppm/tabelas (for livestock production) and https://sidra.ibge.gov.br/pesquisa/pam/tabelas (for crop production). Then, based on the Brazilian Table of Food Composition (TACO) [3], we converted the agricultural production to nutrient equivalent (Equation 1). Whenever a specific nutritional composition was not reported in TACO, we used the United States Department of Agriculture (USDA)'s National Food and Nutrient Analysis Program (NFNAP) values [4].

(1) nutrientproduction=agriculturalproduction*nutritionalcomposition

Where, *nutrient production* is the nutritional equivalent production of one of the nutrients in a given municipality and year (units per 100g of product are reported in the columns of Table 1) from each the commodities in a given municipality and year (*agricultural production* in tonnes of each product as reported by the IBGE, listed in the columns of Table 1) and *nutritional composition* is the value of each nutrient for each commodity, as shown in Table 1.

The amount of nutrients produced in each municipality was calculated by summing the nutritional content of each crop and livestock production in each year (Equation 2).

(2) totalnutrientproduction=Σnutrientproduction

Where, *total nutrient production* is the sum of a nutrient produced by all of the 62 crops and 5 livestock commodities in a given municipality and year. This database provide a specific table for each nutrient, as described in the previous section (20 tables named with the specific nutrient).

### 2.2 Allocation of the nutrients according to the Food Balance Sheets

Then, we allocated the nutrients into “Processed”, “Food”, “Export”, “Feed”, “Seed”, “Other Uses” and “Losses” based on the FAO's FBS, that report the production and amount of the commodities that are distributed into these categories. We did not use the raw values of the FBS, but the proportion of them (relative to the total production reported in the FBS) because the FAO's data is reported in 1000 units of the IBGE's units: tonnes of crop, kilograms of meat (beef, pig and poultry), litres of milk or thousand dozens of eggs. We thus opted to preserve the more detailed information reported by the IBGE.

The allocation into the FBS's categories followed the Eq. 3:

(3) Allocation=nutrientproduction*FBSproportion

Where, *Allocation* is the FBS's categories described above, *nutrient production* is derived from Equation 1 and *FBS proportion* is the proportion of each category reported in the FBS, relative to the total production from FBS. For each nutrient in a specific table, the *Allocation* is provided for all of the municipalities and years in eight rows in the column called “FBS” in the dataset.

As in reference [2], some assumptions had to be made in order to allocate the nutrients into FAO's categories: “Other Uses” of Cottonseed were considered as “Fibres” (thus, lost from the food system) and “Other Uses” of potatoes and cassava, which were 2% and 6% of the total production in the period, were allocated to “Food”, as they are staple food in Brazil. Also, the amount of “Other Uses” of Milk was allocated to “Food”, as it might represent cheese, yoghurt and other dairy products for human consumption. In the case of Sugar-Cane, “Other Uses” corresponds to the ethanol production for biofuel, while the “Processed” portion matches the national sugar production. “Processed” proportions of Castor Beans, Cottonseed and Soybeans were allocated to Oil while the “Processed” proportions of Groundnut, Rice, Barley, Coconuts, Maize and Grapes were allocated proportionally as food or feed, according to the domestic use. The ratio of Oil or Ethanol production to “Processed” crop (e.g. soybeans or sugar cane) from FBS was used to convert raw crop nutrients to Oil or Ethanol. In the case of soybeans, the range of fraction of crop production that is converted to Oil is between 0.17 and 0.20 in the time series, while the fraction of sugar cane ranges from 0.10 to 0.13. Once the “Processed” calories were calculated, they were allocated to “Export”, “Feed” and “Food” according to the respective proportion of these categories in the Oil and Sugar sheets. FAO's Technical Conversion Factors for Agricultural Commodities [5] was used to convert livestock weight to edible proportions of beef, pig and poultry meat.

Since crop and livestock production were available in Brazil at sub-national level until 2017 and the FBS only until 2013, by the time we built this database, we modelled the proportions of food, feed, exports etc. for the period of 2014 to 2017 using linear regression with IBGE's production as the predictor. To minimize the effects of the trend in the time series, and to ensure that the best regression parameters were used, for each of the agricultural products we ran 24 different regressions comprising different periods, i.e. 1988-2013, 1989-2013, 1990-2013… 2011-2013, and the model with the higher adjusted R² was selected to predict food, feed, exports and processed products. The adjusted R² was used for model selection because it takes into account the sample size, thus it was a suitable parameter to compare the models.

The “Food”, “Export”, “Feed”, “Seed”, “Other Uses” and “Losses” rows in the tables also account for the processed products, thus when summing these 6 rows the result is equal to the total nutrient production in a given municipality. The row “Processed” should not be considered in this case to avoid double counting of the nutrients. “Losses” also include nutrient loss in the conversion of raw crop to oil or sugar.

## Declaration of Competing Interest

The authors declare that they have no known competing financial interests or personal relationships which have, or could be perceived to have, influenced the work reported in this article.

## Appendix Supplementary materials

Image, application 1
